# Human dendritic cell maturation induced by amorphous silica nanoparticles is Syk-dependent and triggered by lipid raft aggregation

**DOI:** 10.1186/s12989-023-00527-9

**Published:** 2023-04-19

**Authors:** Éléonore Guillet, Émilie Brun, Céline Ferard, Kévin Hardonnière, Myriam Nabhan, François-Xavier Legrand, Marc Pallardy, Armelle Biola-Vidamment

**Affiliations:** 1 INSERM UMR-996, Inserm, Inflammation, Microbiome and Immunosurveillance, Faculté de Pharmacie, Université Paris-Saclay, 17, Avenue Des Sciences, 91400 Orsay, France; 2 Institut de Chimie Physique, CNRS, Université Paris-Saclay, 91400 Orsay, France; 3 Institut Galien Paris-Saclay, CNRS, Université Paris-Saclay, 91400 Orsay, France

**Keywords:** Amorphous silica, Nanoparticles, Dendritic cells, Syk, Lipid rafts, Src kinases

## Abstract

**Background:**

Synthetic amorphous silica nanoparticles (SAS-NPs) are widely employed in pharmaceutics, cosmetics, food and concretes. Workers and the general population are exposed daily via diverse routes of exposure. SAS-NPs are generally recognized as safe (GRAS) by the Food and Drug Administration, but because of their nanoscale size and extensive uses, a better assessment of their immunotoxicity is required. In the presence of immune “danger signals”, dendritic cells (DCs) undergo a maturation process resulting in their migration to regional lymph nodes where they activate naive T-cells. We have previously shown that fumed silica pyrogenic SAS-NPs promote the two first steps of the adaptative immune response by triggering DC maturation and T-lymphocyte response, suggesting that SAS-NPs could behave as immune “danger signals”. The present work aims to identify the mechanism and the signalling pathways involved in DC phenotype modifications provoked by pyrogenic SAS-NPs. As a pivotal intracellular signalling molecule whose phosphorylation is associated with DC maturation, we hypothesized that Spleen tyrosine kinase (Syk) may play a central role in SAS-NPs-induced DC response.

**Results:**

In human monocyte-derived dendritic cells (moDCs) exposed to SAS-NPs, Syk inhibition prevented the induction of CD83 and CD86 marker expression. A significant decrease in T-cell proliferation and IFN-γ, IL-17F and IL-9 production was found in an allogeneic moDC:T-cell co-culture model. These results suggested that the activation of Syk was necessary for optimal co-stimulation of T-cells. Moreover, Syk phosphorylation, observed 30 min after SAS-NP exposure, occurred upstream of the c-Jun N-terminal kinase (JNK) Mitogen-activated protein kinases (MAPK) and was elicited by the Src family of protein tyrosine kinases. Our results also showed for the first time that SAS-NPs provoked aggregation of lipid rafts in moDCs and that MβCD-mediated raft destabilisation altered Syk activation.

**Conclusions:**

We showed that SAS-NPs could act as an immune danger signal in DCs through a Syk-dependent pathway. Our findings revealed an original mechanism whereby the interaction of SAS-NPs with DC membranes promoted aggregation of lipid rafts, leading to a Src kinase-initiated activation loop triggering Syk activation and functional DC maturation.

**Supplementary Information:**

The online version contains supplementary material available at 10.1186/s12989-023-00527-9.

## Background

If many nanoparticles are generated from natural sources such as fires, volcanic eruptions, oceans, and groundwater, engineered nanoparticles have been increasingly used in electronics, cosmetics, food, and pharmaceuticals over the past decades [1]. Synthetic amorphous silica nanoparticles (SAS-NPs) are among the three most-produced nanomaterials [2] obtained either by a wet process for precipitated silica or by high-temperature flame pyrolysis for pyrogenic silica [3]. Fumed silica is found in various daily used products such as cosmetics as an anticaking agent or dehydrated food as the E551 additive [4, 5]. The manufacturing and industrial uses raise the issue of occupational exposure, mainly occurring through the respiratory route and enabling contact with immune cells in the respiratory tract [6], such as macrophages or dendritic cells (DCs). SAS-NPs have been described as Generally Recognized As Safe (GRAS) by the Food and Drug Administration and have long been considered biocompatible compared with crystalline silica. However, recently we and others have found that SAS-NPs may trigger immune cells leading to immunotoxic effects [7, 8].

DCs are professional antigen-presenting cells (APCs) with phagocytic capabilities [9]. They sample their environment and behave as immunological sentinels [10]. Indeed, DCs reside in non-lymphoid tissues in an immature antigen-capturing state until a danger signal warns them that their microenvironment is harmful [11, 12]. A maturation process is consequently engaged, allowing DC migration to regional lymph nodes. Exogenous danger signals, known as Pathogen-Associated Molecular Patterns (PAMPs), are evolutionary conserved common structures shared by many pathogens but not found in their eukaryotic hosts [13]. Endogenous danger signals, also called alarmins or Damage-Associated Molecular Patterns (DAMPs), are constitutive or inducible and released by cells or tissues undergoing non-physiological stress or death [13]. More recently, a new danger category has been proposed, the Nanoparticle-Associated Molecular Patterns (NAMPs) [14], which depend on both the physicochemical properties of the particles and the nature of the components interacting with them when they encounter biological fluids [15]. Observations from both in vivo and in vitro studies support the hypothesis of SAS-NPs being a danger signal. Indeed, upon repetitive inhalation exposure, a reversible inflammation was evidenced in rodents, associated with IL-1ß production [16, 17]. In mice, SAS-NPs could act as adjuvants in allergic responses when the nanoparticles are administered by intranasal instillation [18, 19]. Furthermore, intraperitoneal exposure to SAS-NPs showed an adjuvant effect in a model of ovalbumin-induced allergy in mice [20]. This was demonstrated by higher anti-OVA IgG1, IgG2a, IgE antibody production, and enhanced cytokine production (IFN-γ, IL-2, IL-4, IL-5, IL-17) [20]. Moreover, in vitro SAS-NPs trigger an activation of murine DCs reflected by an increased expression of the CD80 and CD86 markers and the major histocompatibility complex-II (MHC-II) as well as IL-1ß [21] and TNF-α pro-inflammatory cytokine production [22]. Vallhov et al. showed that mesoporous SAS-NPs increase the expression of CD86 on human monocyte-derived dendritic cells (moDCs) [23]. Besides, our previous results have shown that SAS-NPs increased the expression of CD83, CD86 and CXCR4 markers as well as the secretion of pro-inflammatory cytokines (CXCL-8 and CXCL-12) by moDCs. Fumed silica-activated DCs significantly increased human T-lymphocyte proliferation in an allogeneic co-culture model [24, 25]. Taken together, these observations strongly support that SAS-NPs could behave as immunogenic danger signals. However, there is very limited data so far on the early events initiated by the interaction of nanomaterials with DCs and the signalling pathways that could be turned on and thus participate in the activation of these cells.

Syk is a central protein kinase in intracellular signalling known to be involved in DC maturation following the engagement of receptors or direct interactions of particles with the plasma membrane [26, 27, 28]. The initial activation of Syk is mainly linked to its phosphorylation by Src family kinases, initiating a phosphorylation loop [29, 30]. Sedlik et al. have shown that Syk plays a crucial role in the signalling process following FcγR binding, which leads to the induction of DC maturation [31]. This has also been demonstrated in response to therapeutic protein aggregates following their interaction with FcγR [28]. Syk is involved in DC maturation through the activation of signalling cascades such as Rac GTPase pathways, involved in c-Jun N-terminal kinase (JNK) activation but also in phagocytosis and antigen internalisation [32, 33]. The activation of Syk and the signalling pathways engaged following nanomaterial interactions with DCs have not been investigated yet. However, studies on monosodium urate crystals (MSU) and alum particles showed that Syk could be phosphorylated following MSU direct membrane binding leading to cell surface lipid sorting [26, 34]. A similar phenomenon has been demonstrated with cholesterol crystals [27]. Although little is known about the early activation of the Syk axis following nanomaterial interactions with DCs, it is well established that Syk is at the crossroads of several subjacent signalling pathways such as Phosphoinositide 3-kinases (PI3K) or Mitogen-activated protein kinases (MAPK).

The purpose of this work was to investigate whether Syk fulfils a role in DC activation in response to SAS-NPs and to identify the mechanisms of Syk activation following the interaction of DCs with SAS-NPs. In this study, we identified the initial events leading to Syk activation in human DCs in response to SAS-NPs. We demonstrated that SAS-NPs trigger lipid raft aggregation leading to Src kinase recruitment, initiating the autophosphorylation loop of Syk and resulting in the phosphorylation of JNK. Taken together, SAS-NP activation of this signalling pathway induces DC maturation.

## Results

### Characterization of SAS-NPs

We used fumed silica SAS-NPs supplied by Sigma-Aldrich (S5505, Sigma-Aldrich, St Quentin Fallavier, France), selected as a model of silicas of industrial origin. This material showed comparable results with the NM-202 benchmark material from the Joint research center repository on moDC phenotype, inflammatory cytokine and chemokine secretion [7]. The silica nanopowder is presented by the manufacturer as having an average primary particle size of 14 nm and a specific surface area of 200 ± 25 m^2^.g^−1^. A precise characterisation of the SAS-NPs used in this study has been previously reported [7]. The main characteristics are summarized in Table 1.

**Table 1:** Characteristics of SAS-NPs

Reference	Specific surface area^1^	Nominal primary particle diameter^1^	Primary particle diameter^2^	DLS distribution^3^	ζ-potential value^3^
S5505, Sigma-Aldrich	196 m^2^.g^−1^	14 nm	14.4 ± 5.3 nm	123 ± 53 nm 8.7 ± 0.3 µm	− 22.5 ± 1.4 mV

^1^Data provided by the supplier, ^2^ Analyzed by TEM, ^3^ in RPMI 1640

### Syk controls moDC phenotype in response to SAS-NPs

**Fig. 1:**
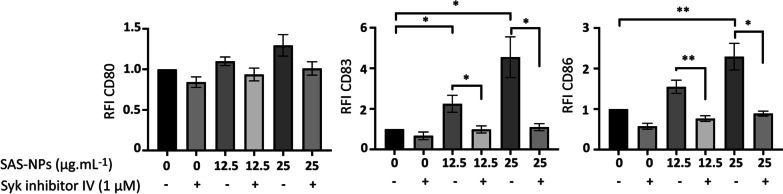
Syk controls moDC phenotype in response to SAS-NPs. Immature moDCs were pre-treated for 1 h with Syk inhibitor IV (1 μM) and then treated for 16 h with SAS-NPs (12.5 or 25 μg.mL^−1^). Cells were then collected, washed, and analyzed by flow cytometry for surface marker expression. Results are expressed as relative fluorescence intensity (RFI) compared with non-stimulated cells and represented as the mean ± SEM of 3 independent experiments. *p < 0.05; **p < 0.01 One-way Anova, Kruskal–Wallis test

Syk is a well described tyrosine kinase involved in the control of signalling pathways in innate immune cells [28]. To evaluate whether Syk was involved in the response of moDCs to SAS-NPs, we used a well-described highly selective pharmacological inhibitor of Syk, the Syk Inhibitor IV (BAY 61–3606) (Fig. 1). Cells were pre-treated for one hour with Syk Inhibitor IV and then stimulated with SAS-NPs for 16 h. The inhibitor’s concentration was optimised to achieve effective inhibition and to avoid cellular toxicity [28]. In the absence of Syk inhibitor IV, SAS-NPs induced a significant increase in the CD83 and CD86 marker expression, at 12.5 and 25 µg.mL^−1^ for CD83 and only at 25 µg.mL^−1^ for CD86. The inhibitor completely prevented CD83 and CD86 expression provoked by SAS-NPs, both at 12.5 or 25 µg.mL^−1^ (Fig. 1). This result showed that Syk plays a major role in SAS-NPs-induced DC maturation.

### Activation of Syk in moDCs controls allogeneic T-cell proliferation and cytokine production induced by SAS-NP-treated moDCs

**Fig. 2:**
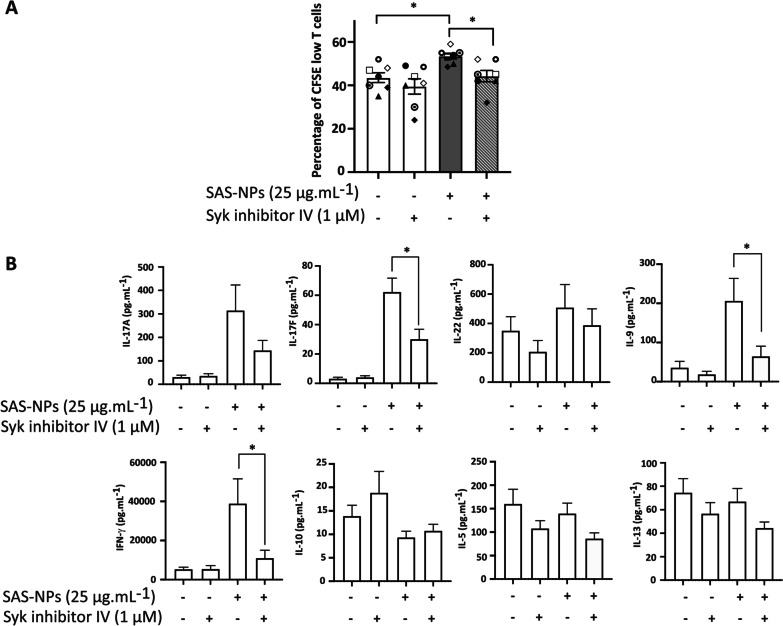
Syk inhibition alleviates allogeneic T-cell response to SAS-NP-treated moDCs. Immature moDCs were pre-treated for one hour with Syk inhibitor IV (1 μM) and then treated for 16 h with 25 µg.mL^−1^ of SAS-NPs. Treated moDCs were co-cultured with allogeneic CD4^+^ T-cells loaded with CFSE at a ratio of 1 moDC for 20 CD4^+^ T-cells. **A** Proliferation was measured after 5 days of co-culture as the percentage of CFSE^low^ CD4^+^ T-cells. Untreated moDCs were used as control. **B** On day 5, cytokine levels were quantified in co-culture supernatants in duplicate, using an electroluminescent multiplex assay. Detection limits are indicated in the Methods section. Results for lymphocyte proliferation are expressed as percentage of CFSE low cells. Cytokine production is expressed in pg.mL^−1^. Both are represented as the mean ± SEM of 6 independent experiments. *p < 0.05 One-way Anova and Kruskal–Wallis test

To assess the role of Syk activation in T-cell proliferation, we used an allogeneic moDC:T-cell co-culture model. MoDCs were pre-treated for one hour with Syk Inhibitor IV and then stimulated or not with SAS-NPs. T-cell proliferation was assessed on day 5 as the percentage of CFSE^low^ CD4^+^ T-cells (Fig. 2A). Using a 1:20 DC:T-cell ratio as previously reported [24], non-treated allogeneic moDCs induced a basal T-cell proliferation (43.5%) that was statistically increased upon exposure of moDCs to SAS-NPs (50%) (Fig. 2A). This result showed that SAS-NP-induced moDC phenotype modification resulted in an augmentation of T-cell proliferation. Interestingly, the inhibition of Syk in SAS-NP-treated cells caused a significant decrease in the percentage of CFSE^low^ CD4^+^ T-cells compared with SAS-NP-activated moDCs, suggesting that Syk activation was essential for full DC maturation and co-stimulation of T-cells (Fig. 2A). We have previously demonstrated that SAS-NPs increased the production of IL-9 and IL-17 A/F by CD4^+^ T-cells compared to untreated moDCs [7]. We therefore assessed whether Syk could contribute to cytokine production by CD4^+^ T-cells in response to SAS-NPs. To this end, we measured cytokines in the moDC:CD4^+^ T-cell co-culture supernatants presented in Fig. 2A. Notably, we detected a significant decrease of IFN-γ, IL-17F and IL-9 production following Syk inhibition (Fig. 2B). Furthermore, the production of IL-10, IL-5, IL-13, IL-17A and IL-22 was not affected. These results reinforce the conclusion that the activation of Syk in moDCs following SAS-NP treatment would be necessary for the establishment of the T-cell response.

### Syk is activated in SAS-NP-treated moDCs

**Fig. 3:**
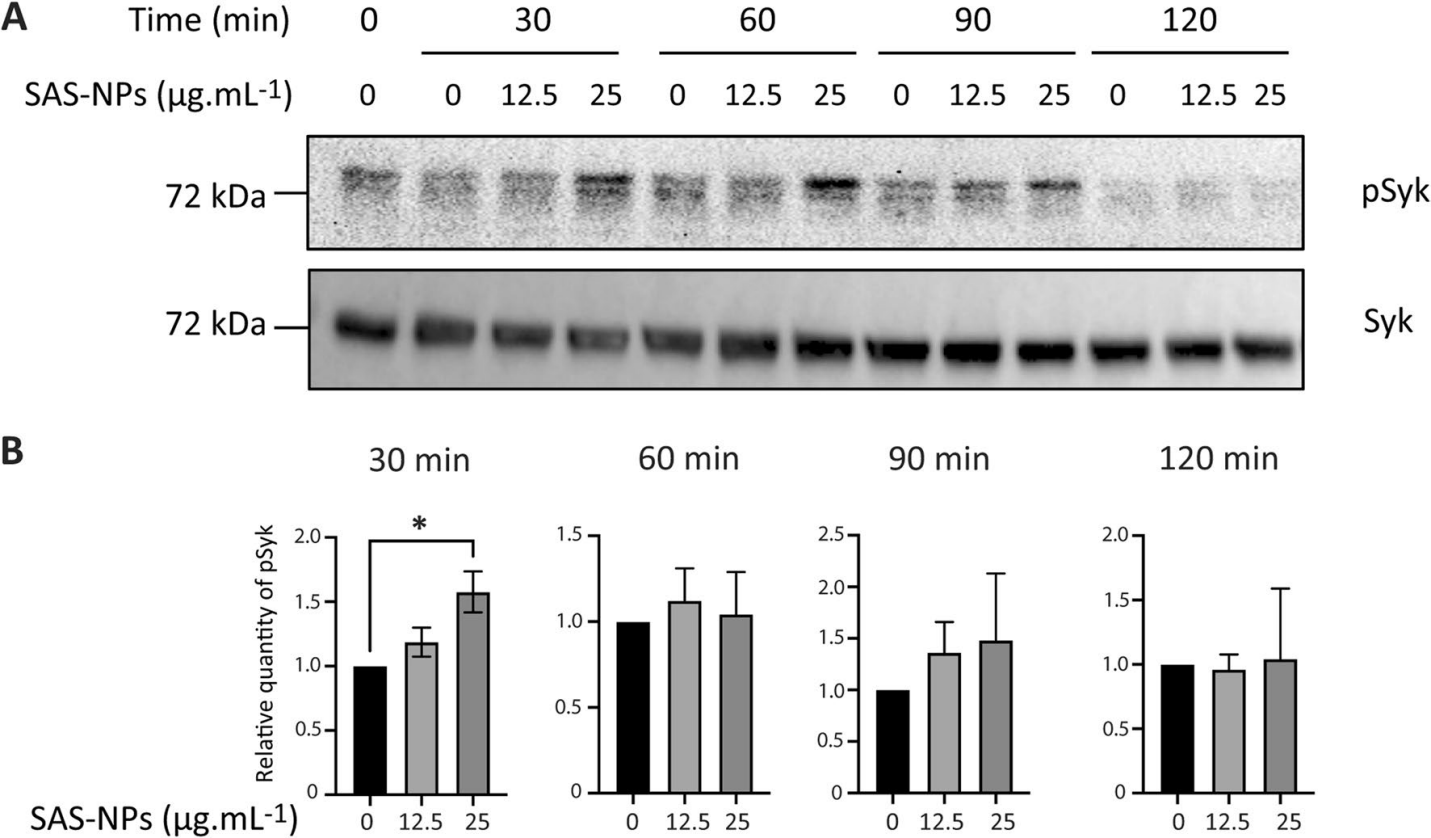
Syk is phosphorylated in response to SAS-NPs. Immature moDCs were treated for 30, 60, 90 and 120 min with SAS-NPs (12.5 or 25 μg.mL^−1^). Immunoblotting of whole-cell extracts was used to quantify the phosphorylated form of Syk. **A** Representative experiment. **B** Results of 3 independent experiments are represented. Bands were quantified using the Image Lab software. Results are expressed as the fold induction, representing the ratio of the normalised intensity of specific bands of treated cells divided by the normalised intensity of bands of untreated cells (pSyk/Syk). *p < 0.05 One-way Anova, Kruskal–Wallis test

To characterise Syk activation in moDCs upon SAS-NP exposure, we next measured the kinetics of Tyr 525/526 phosphorylation in the activation loop of the Syk kinase domain using Western Blotting (Fig. 3). Despite the inherent inter-individual variability between donors, we reproducibly detected a phosphorylation of Syk starting at 30 min after stimulation with SAS-NPs (Fig. 3A). Interestingly, at that very time, Syk phosphorylation increased in a concentration-dependent manner and was significantly different from the control at the highest concentration of 25 µg.mL^−1^ (Fig. 3B). This phosphorylation was maintained or not, depending on the donors, at 60 min, becoming undetectable at 120 min.

**Fig. 4:**
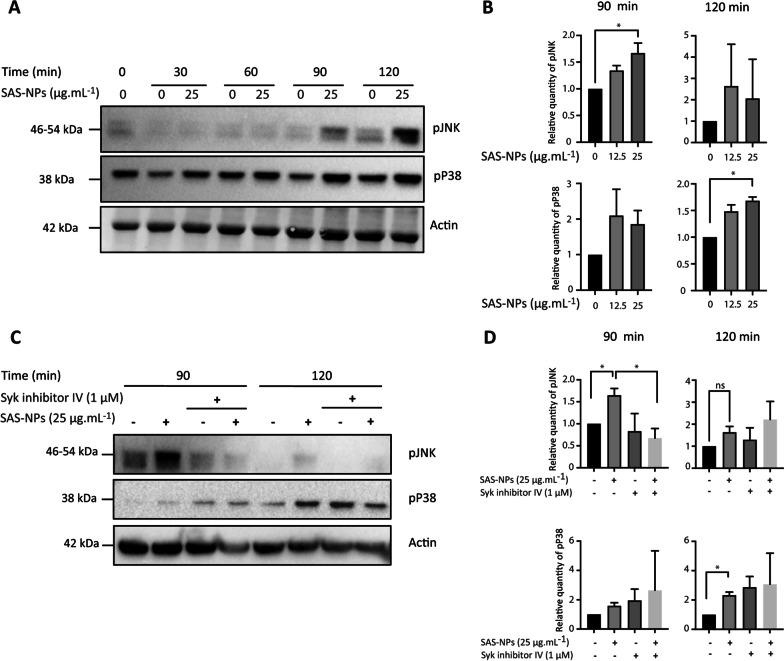
JNK and p38 MAPK are phosphorylated in response to SAS-NPs. A and B. Immature moDCs were treated for 30, 60, 90 and 120 min with SAS-NPs (12.5 or 25 μg.mL^−1^). **A** Representative experiment. **B** Immunoblotting of whole-cell extracts was used to quantify the phosphorylated forms of JNK and p38 kinases. C and D. Immature moDCs were pre-treated for 1 h with Syk inhibitor IV (1 μM) and then treated for 90 or 120 min with 25 µg.mL^−1^ of SAS-NPs. **C** Representative experiment. **D** Immunoblotting of whole-cell extracts was used to quantify the phosphorylated forms of JNK and p38. The results of 3 independent experiments are represented. Bands were quantified using the Image Lab software. Results are expressed as the fold induction, representing the ratio of the normalised intensity (pJNK/actin or pP38/actin) of specific bands of treated cells divided by the normalised intensity of bands of untreated cells, *p < 0.05 One-way Anova, Kruskal-Wallis test.

Syk is a protein tyrosine kinase upstream of many signalling pathways such as MAPK [27, 35] which have been described to be involved in moDC maturation [36, 37, 38, 39, 40]. To evaluate whether MAPKs also contribute to the response of moDCs to SAS-NPs, we assessed their phosphorylation using Western blotting (Fig. 4A). Two MAPKs, JNK and p38, were found to be significantly activated at 90 and 120 min after SAS-NP addition respectively (Fig. 4B). These results show that the MAPK pathway is activated following the interaction between moDCs and SAS-NPs, but this delayed phosphorylation suggests an indirect activation where an upstream signalling pathway would be turned on first. To ascertain whether Syk is required for MAPK activation in response to SAS-NPs, we measured JNK and p38 phosphorylation upon Syk inhibition. Cells were pre-treated for one hour with Syk Inhibitor IV and then stimulated for 90 or 120 min with SAS-NPs (Fig. 4C). A significant decrease in JNK phosphorylation in response to SAS-NPs was observed while no effect on p38 phosphorylation was detected (Fig. 4D).

Taken together, our results indicated that phosphorylation of Syk in response to SAS-NPs occurred upstream of JNK but not p38 MAPK. Moreover, the phosphorylation of these two kinases appeared to be triggered by SAS-NPs.

### Src family protein kinases play a role in Syk phosphorylation in response to SAS-NPs

**Fig. 5:**
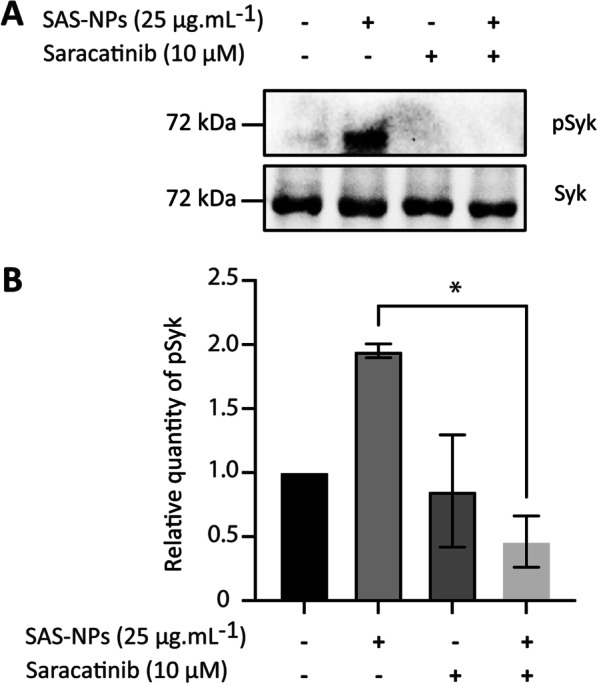
Src kinases are required to initiate Syk phosphorylation in response to SAS-NPs. Immature moDCs were pre-treated for 1 h with Saracatinib (10 µM) and then treated for 30 min with 25 µg.mL^−1^ of SAS-NPs. **A** Representative experiment. **B** Immunoblotting of whole-cell extracts was used to quantify the phosphorylated form of Syk. The results of 3 independent experiments are represented. Bands were quantified using the Image Lab software. Results are expressed as the fold induction, representing the ratio of the normalised intensity of specific bands of treated cells divided by the normalised intensity of bands of untreated cells, *p < 0.05 One-way Anova, Kruskal–Wallis test

As previously stated, activation of Syk can result from the Syk activation loop, initiated by Src family kinases and then relayed and amplified by other activated Syk molecules [29]. We therefore assessed whether Syk phosphorylation observed in response to SAS-NPs could be induced by a Src family protein kinase. To this end, cells were pre-treated for one hour with the Src kinase inhibitor Saracatinib (Lyn, Fyn and Lck inhibitor) and then stimulated for 30 min with SAS-NPs (Fig. 5A). The inhibitor’s concentration was optimised to achieve effective inhibition and to avoid cellular toxicity [28].

Results showed a significant decrease in Syk phosphorylation in response to SAS-NPs when using Saracatinib (Fig. 5B). This result highlights that Src family kinases were involved in the phosphorylation of Syk in response to SAS-NPs.

### Involvement of lipid rafts in SAS-NP-treated moDCs

**Fig. 6:**
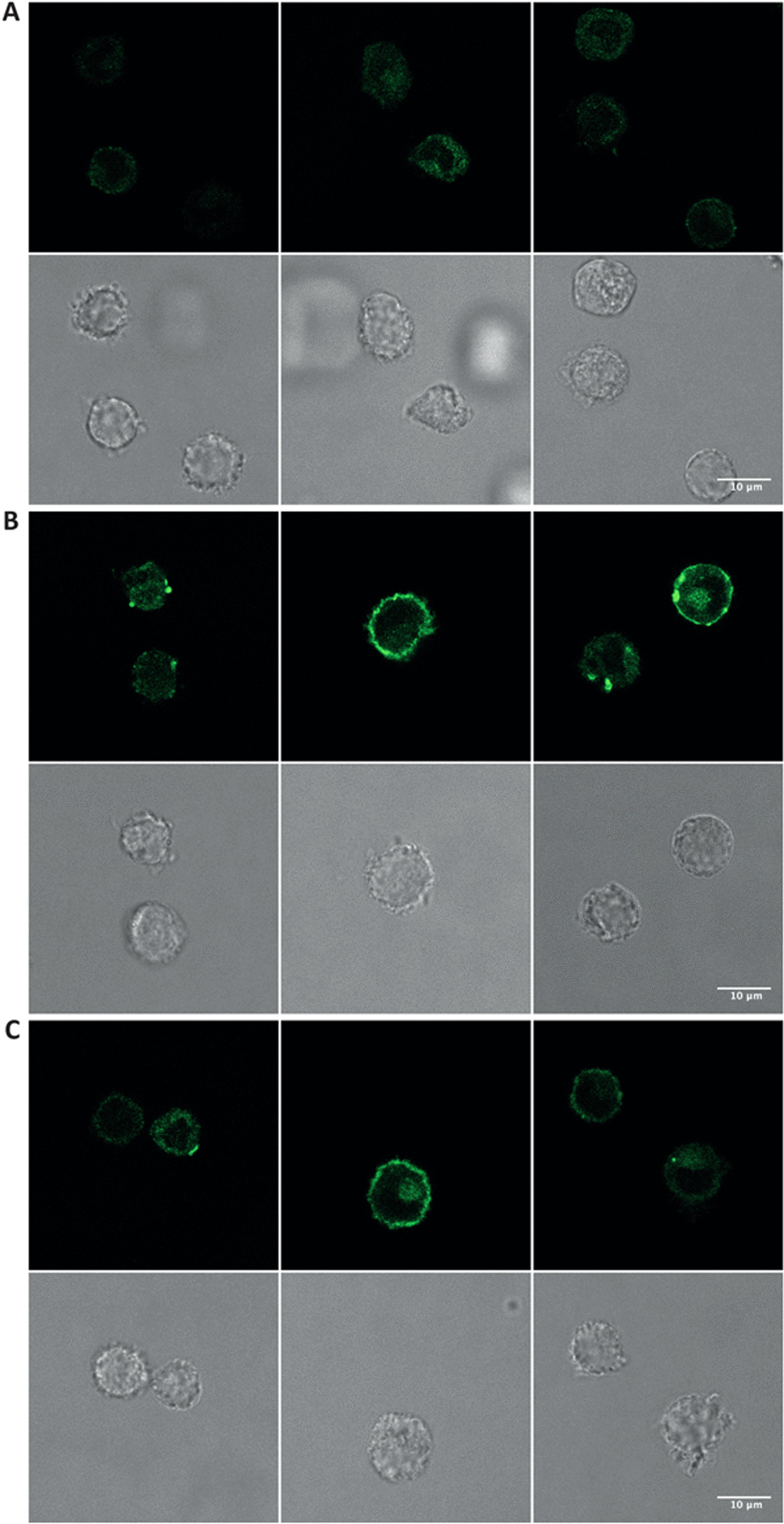
SAS-NPs induce lipid raft aggregation in moDCs. Visualisation of lipid rafts by conventional fluorescence microscopy using cholera toxin subunit B conjugated with Alexa Fluor 488 which binds to the raft-associated monosialotetrahexosylganglioside (GM1). Immature moDCs were treated for 15 (**B**) or 30 min (**C**) with SAS-NPs (25 μg.mL^−1^) or left untreated (**A**) and then stained with cholera toxin subunit B conjugated with Alexa Fluor 488. The experiment was replicated twice. Left/middle/right panels: 3 different fields of the same experiment

Lipid rafts are cell membrane microdomains enriched in cholesterol and sphingolipids forming liquid-ordered domains of decreased membrane fluidity. Importantly, lipid rafts represent a supramolecular platform for cellular signalling events bringing together receptors, channels and concentrating proteins in the inner face of the plasma membrane as found for the dually acylated Src kinases Lck, Fyn and Lyn [41, 42]. Since the post-translational acylations and association with lipid rafts have been reported to be critical in the regulation and activation of Src kinases [43], we hypothesised that an early effect on the plasma membrane leading to lipid raft aggregation could be involved in SAS-NP-elicited Syk phosphorylation. As a first step, using an Alexa Fluor conjugate of the cholera toxin B subunit to label the monosialotetrahexosylganglioside (GM1), a well-known marker of lipid rafts, we assessed whether the interaction of moDCs with SAS-NPs could trigger lipid raft formation (Fig. 6).

In untreated moDCs, as expected, GM1 featured a diffuse distribution (Fig. 6A). The staining of the raft-associated GM1 glycosphingolipid showed that SAS-NPs led to visible raft aggregation starting 15 min after treatment (Fig. 6B), which seemed to be lowered but still observable after 30 min (Fig. 6C). These timepoints appear to be consistent with the activation sequence of Syk (significant after 30 min of stimulation, Fig. 3).

**Fig. 7:**
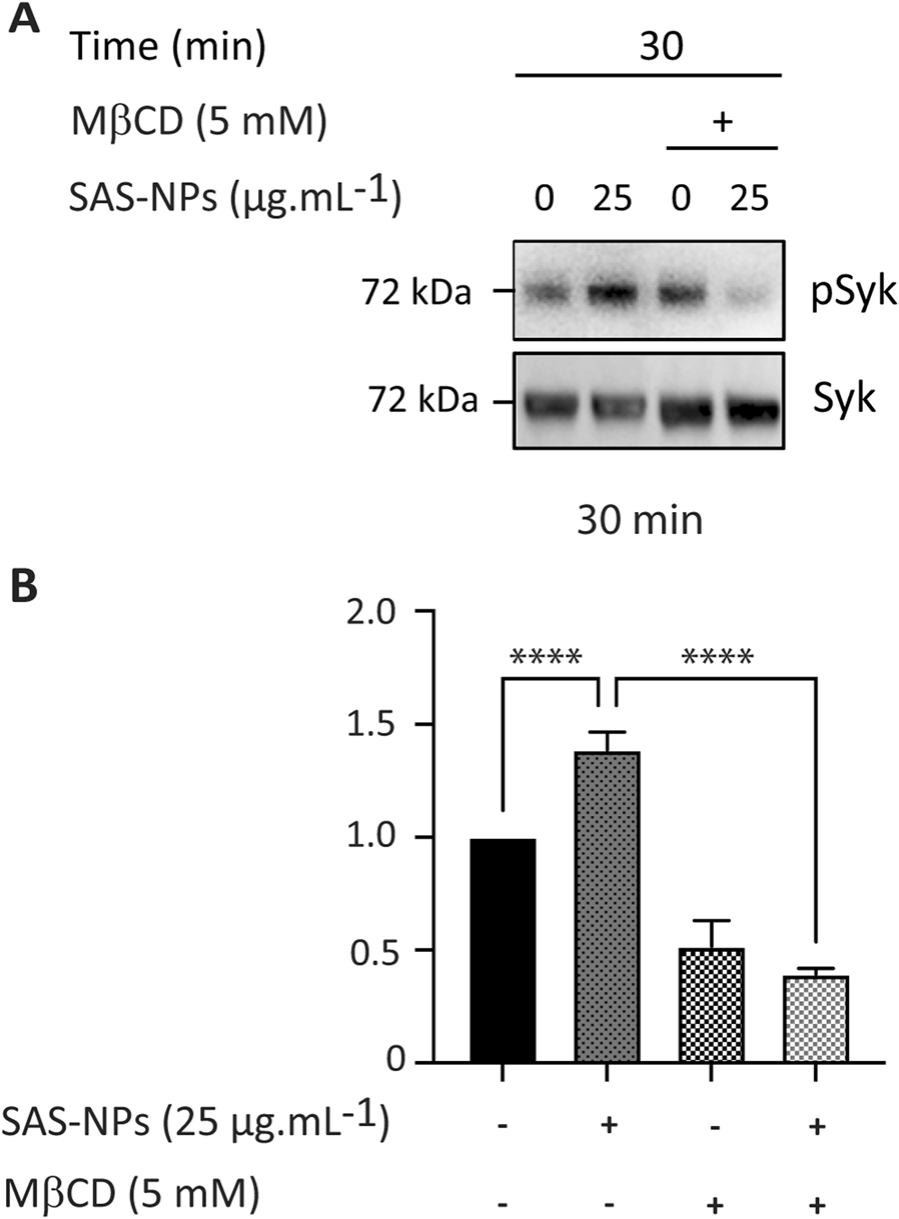
Lipid rafts are necessary to activate Syk in response to SAS-NPs. Immature moDCs were pre-treated for 1 h with Methyl-ß-cyclodextrin (MßCD, 5 mM) and then treated for 30 min with 25 µg.mL^−1^ SAS-NPs. **A** Representative experiment. **B** Immunoblotting of whole-cell extracts was used to quantify the phosphorylated form of Syk. The results of 3 independent experiments are represented. Bands were quantified using the Image Lab software. Results are expressed as the fold induction, representing the ratio of the normalised intensity of specific bands of treated cells divided by the normalised intensity of bands of untreated cells, ****p < 0.0001 One-way ANOVA and Bonferroni’s multiple comparison test

To further support our observation, we then investigated whether Syk activation following SAS-NP exposure could occur under conditions of lipid raft destabilisation. Cholesterol plays a key structural role in the microdomain architecture and the use of cholesterol-sequestering drugs to disrupt membrane rafts is now well established [44]. As a second step, we therefore used methyl-ß-cyclodextrin (MßCD) to extract cholesterol from the membranes and investigated the consequences of such a cholesterol depletion on Syk activation by SAS-NPs (Fig. 7).

After 30 min of SAS-NP treatment, cholesterol depletion resulted in undetectable Syk phosphorylation (Fig. 7A). Moreover, at this time, Syk phosphorylation was significantly decreased compared to the control without MβCD (Fig. 7B). Together, these results indicated that SAS-NPs induced lipid raft formation and that these rafts were required for the activation of Syk in response to SAS-NPs.

## Discussion

DCs are sentinels of the immune system, actively sampling their antigenic environment and sensing danger. Some nanomaterials have been shown to activate DCs, either directly or as a result of cell injury or death, and could thus be considered as an emerging category of danger signals [45]. We have previously demonstrated that it applies to SAS-NPs, inducing efficient DC maturation and pro-inflammatory cytokine release [7]. As a further step in this work, we describe here, to our knowledge for the first time, that SAS-NPs induce DC maturation by promoting lipid raft formation and the subsequent activation of a signalling cascade involving sequentially Src, Syk kinases and MAPKs, consistent with a functional maturation process.

One of the most critical features of DC biology is their functional immunogenic maturation enabling antigen processing and presentation, and T-cell co-stimulation. First, we showed that the response of DCs and T-cells to SAS-NPs was Syk-dependent. Upon inhibition of Syk, CD83 and CD86 molecules were significantly decreased in response to SAS-NPs. Moreover, Syk was also found to be required for T-cell co-stimulation as well as IL-17A, IL-9 and IFN-γ release using an allogeneic DC:T-cell co-culture model. In the literature, the contribution of Syk to DC maturation has been documented [31] following therapeutic protein aggregates, MSU or alum crystals exposure [26, 28, 34].

Syk is known to be activated upon receptor engagement such as the classical immunoreceptors: the T cell receptor (TCR) [46], the B cell antigen receptor (BCR) [47], the receptor for the Fc region of IgG (FcγR), as well as the C-type lectin receptor Dectin-1 or the Macrophage inducible Ca2^+^-dependent lectin receptor Mincle [48]. Receptor ligation and clustering of the ITAM (immunoreceptor tyrosine-based activation motif) in Fc receptor complexes [46] result in the rapid phosphorylation of ITAM tyrosine motif, primarily by Src kinases. Syk is then recruited to the membrane by binding to the doubly phosphorylated ITAM, making it accessible for phosphorylation by Src kinases and thus initiating an autophosphorylation loop [49]. Syk activation is commonly detected only a few minutes after the interaction between the ligand and its specific receptor [28, 50]. However, in our study, we observed a rather late activation of Syk after 30 min of stimulation, in line with a receptor-independent process. Interestingly, earlier stimulation timepoints were analysed in a preliminary experiment, but no phosphorylation was observed (data not shown). According to the literature, Syk is known to play a central role upstream of many signalling pathways suck as the MAPKs [49]. We found that JNK and p38 MAPK were activated at the late timepoints of 90 and 120 min respectively. In many cellular models, phosphorylation of MAPKs is observed as early as 15 min or even earlier [38, 51, 52]. These results are in line with the observation of delayed Syk activation. MAPK activation by SAS-NPs is also consistent with the literature, where MAPKs have been widely involved in DC maturation in response to various *stimuli* such as chemical sensitisers (nickel, cobalt or dinitrochlorobenzene) [38, 40] and also to Sigma “ultrafine” silica particles [22]. Surprisingly, Syk was required for JNK phosphorylation but not for p38 MAPK. It has already been reported in rat vascular smooth muscle cells that both JNK and p38 MAPK signalling pathways could be independently activated in response to the same stimulus, since only the activation of JNK but not of p38 MAPK was mediated by Src kinases in presence of hydrogen peroxide [53].

The late phosphorylation detected with both Syk and MAPKs was highly suggestive of an indirect activation, involving upstream events unlikely to result from a receptor engagement. According to the literature, Syk activation is thought to occur in an autophosphorylation loop initiated by Src family kinases [29, 54]. Our results showed that Src kinase inhibition altered the phosphorylation of Syk in response to SAS-NPs. By generating the first few molecules of activated Syk, Src kinases would act here as the initiating trigger. Consistent with our findings, it has been shown in antigen-presenting cells such as B cells that Src kinases can be associated with the activation of Syk [55]. 

Aggregation of lipid rafts significantly facilitates many cellular events including signal transduction, endocytosis, vesicular transport and cell adhesion [56, 57]. More specifically, the formation of lipid rafts has been linked to the recruitment of proteins binding glycosylphosphatidylinositol-anchored proteins, transmembrane proteins and doubly acylated protein tyrosine kinases of the Src family [58]. Since their attachment to lipid rafts allows the spatial activation of membrane-anchored Src kinases signalling [59], we wondered whether SAS-NPs could trigger the formation of lipid rafts. Lipid raft aggregation was observed in DCs as early as 15 min after SAS-NP stimulation, consistent with the late phosphorylation of Syk detected after 30 min. When disrupting lipid raft formation using MßCD, Syk phosphorylation induced by SAS-NPs was abrogated. Lipid sorting and then raft reorganisation can occur due to direct membrane interaction with particles. Indeed, Ng et al. have demonstrated in Bone Marrow-Derived Dendritic Cells (BMDC) that solid structures like MSU trigger immune cell activation via membrane lipid alterations without the requirement for specific cell surface receptors [26]. In giant unilamellar vesicles, membrane gelation and decrease of membrane fluidity were reported for oppositely-charged silica NPs [60, 61]. In trout gill epithelial cells, super resolution fluorescence microscopy experiments showed that primary amine-terminated quantum dots preferentially co-localise with lipid rafts [62]. We can therefore hypothesise that such a mechanism supports Src and Syk activation following DC treatment with SAS-NPs.

Recent results from Pavan et al. [63] shed a new light on how SAS-NPs could interact with cell membranes. As lipid rafts are richer in sphingomyelin that exhibits a zwitterionic phosphocholine, their lower local charge allows a more favourable interaction with negatively-charged silica NPs. These authors emphasised the role of nearly-free silanols (NFS), as the main surface pattern responsible for silica particle toxicity [64]. Beside isolated, geminal or vicinal silanols [2], NFS are defined as silanols presenting a very weak mutual interaction through H-bonding. Their inter-distance (4–6 Å) allows the clamping of the phosphate group of phospholipids, that may result in an increase of membrane rigidity. Indeed, in liposome models, these authors observed a restricted motion of the lipophilic fluorescent probe Di-4-ANEPPDHQ, by time-resolved anisotropy measurements [63]. They propose that the membranolytic activity of silica NPs on red blood cells [65, 66] might occur in sphingomyelin-enriched lipid domains, as specific sites of interaction between membranes and NFS. Among all the different particles studied, pyrogenic amorphous silica presents the highest percentage of exposed NFS (17.3%). Such interaction between the cell membrane of DCs and NFS possibly present in the SAS-NPs we studied could stiffen DC membrane and trigger lipid raft aggregation, kinases recruitment and activation and downstream intracellular signalisation.

To conclude, the membrane effects of SAS-NPS could be due to the combination of electrostatic attraction and hydrogen bonding with phospholipids [60]. Among oxide nanoparticles, SAS-NPs seem to cause the most significant changes, including membrane disruption and gelation [60]. These findings could be integrated in parameters accounting for Safe by Design approaches.

## Conclusions

**Fig. 8:**
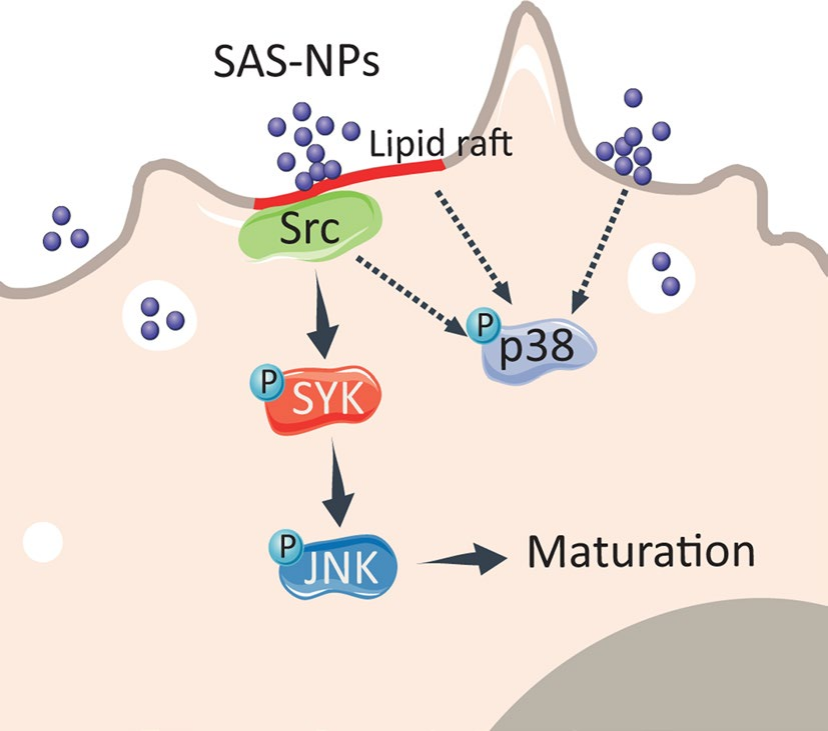
Lipid raft aggregation following SAS-NP exposure leads to Src/Syk signalling pathway activation in human dendritic cells. Upon DC exposure to SAS-NPs, lipid raft aggregation leads to Src kinase recruitment required for the phosphorylation of the tyrosine kinase Syk. Syk phosphorylation is linked to JNK activation and DC maturation. p38 MAPK phosphorylation seems to be independent of Syk. Solid arrows: established links. Dashed arrows: hypotheses. The figure was partly generated using Servier Medical Art, provided by Servier, licensed under a Creative Commons Attribution 3.0 unported license (https://creativecommons.org/licenses/by/3.0/)

In summary, we showed that SAS-NPs were recognised by DCs as a danger signal “like” and triggered DC activation by a Syk-dependent mechanism. The mechanism involved lipid raft aggregation leading to the recruitment of Src kinases, Syk activation which in turn leads to the phosphorylation of JNK and DC maturation (Fig. 8). Taken together, our results provide new insights into the mechanism by which SAS-NPs modify human DC phenotype leading to increased T-cell co-stimulation. We hypothesise that surface chemistry is at the core of the interactions between SAS-NPs and DCs. A more detailed understanding of the interactions between the chemical groups present at the nanoparticle surface and membranes could lead to a safer production of SAS-NPs through a safer-by-design approach.

## Methods

### Synthetic amorphous silica nanoparticles

The fumed silica NPs used in this work are commercially available (S5505, Sigma-Aldrich, St Quentin Fallavier, France, batch SLBR6988V). According to the manufacturer, their specific surface determined by BET (Brunauer–Emmett–Teller) is 196 m^2^.g^−1^. The suspension was prepared from fumed silica dry powder dispersed in filtered ultrapure water at a final concentration of 50 mg.mL^−1^. The suspension was then left for 8 h in a water bath at 80 °C for sterilisation. The absence of bacterial growth in the NP suspension was checked after the process, and a Limulus amebocyte lysate (LAL) test was performed on each batch to address endotoxin contamination. Before each experiment, an intermediate dilution at a concentration of 500 μg.mL^−1^ was prepared in RPMI 1640 medium supplemented with Glutamax, 1 mM sodium pyruvate, 100 µg.mL^−1^ streptomycin, and 100 U.mL^−1^ penicillin (Gibco, Invitrogen, Saint Aubin, France). The suspension was sonicated for 15 min before DC treatment.

### Characterisation of SAS-NPs

The hydrodynamic diameter of particles was determined at 25 °C by Dynamic Light Scattering (DLS) using a Zetasizer Nano ZS 90 (Malvern Instruments, Orsay, France) operating at a fixed scattering angle of 90° and equipped with a Helium–Neon laser source with a wavelength of 633 nm. Measurements were performed in disposable 4-mL cuvettes with a 1-cm optical pathway and four optical faces (Sarstedt, Marnay, France) containing an appropriate volume (1 mL) of the sample, prepared as described above, after dilution to 125 µg.mL^−1^ in the desired medium. The hydrodynamic diameter values were calculated using the Stokes–Einstein equation assuming a spherical shape of the particles. The particle size profile was obtained from the intensity-weighted distribution, and the hydrodynamic diameter value corresponds to the median diameter derived from the cumulative distribution curve. The ζ-potential measurements were carried out with the same instrument at 25 °C by laser doppler velocimetry at a detection angle of 17° in DTS 1060 disposable cells (Malvern Instruments, Orsay, France) loaded with approximately 1 mL of each sample diluted to 375 µg.mL^−1^ in the desired medium. Due to the ionic strength of the samples leading to high conductivity, measurements were performed using the monomodal mode, and the Smoluchowski approximation was used to convert the electrophoretic mobility to ζ-potential. Values are given as mean values of ten measurements. We carried out the characterisation before and after heating at 80 °C. The results (DLS and zeta potential) showed no significant difference, confirming that sterilisation does not alter the physicochemical characteristics of fumed silica NP (Additional file 1: Fig. S1).

### Endotoxin detection

Possible endotoxin contamination was analysed with the Limulus amebocyte lysate (LAL) assay (GenScript, Piscataway, NJ, USA). All batches showed values below the threshold of endotoxin positivity (0.05 EU.mL^−1^).

### Generation of primary cultures of human moDCs

Human monocyte-derived dendritic cells (moDCs) were derived from monocytes isolated from human peripheral blood supplied by the French Blood Bank (Établissement Français du Sang, Rungis, France). Healthy donors gave their written consent for the use of blood donation for research purposes. Peripheral blood mononuclear cells (PBMCs) were sorted from buffy coats by density centrifugation on a Ficoll gradient. Monocytes were then isolated through positive magnetic selection using MidiMacs separation columns and anti-CD14 antibodies coated on magnetic beads (Miltenyi Biotec, Bergisch Gladbach, Germany). Finally, CD14^+^ cells were differentiated in immature moDCs for 4 days in RPMI 1640 supplemented with GlutaMAX (Gibco, Invitrogen), 10% heat-inactivated fetal calf serum (FCS, Gibco), 550 U.mL^−1^ interleukin-4 (rh-IL4, Miltenyi Biotec), 550 U.mL^−1^ GM-CSF (rh-GM-CSF, Miltenyi Biotech), 1 mM sodium pyruvate (Gibco), 100 µg.mL^−1^ streptomycin and 100 U.mL^−1^ penicillin (Gibco) at 37 °C and 5% of CO_2_. On day 4, the differentiation of moDCs was evaluated by flow cytometry. The moDCs used will show the following phenotype: CD83 < 5%, CD86 < 30%, CD1a > 80% and DC-SIGN > 90%. moDCs are then used at the density of 1 million per mL.

### Treatment of moDCs

On day 4, immature moDCs were stimulated from 15 min to 16 h either to 0, 12.5 or 25 µg.mL^−1^ of sonicated SAS-NPs or to 25 ng.mL^−1^ of lipopolysaccharide (LPS, ref: L6529, *Escherichia coli*, serotype 055-B5, Sigma-Aldrich, Saint Quentin Fallavier, France) as a positive control of moDC activation. SAS-NPs were introduced as a concentrated suspension and immediately mixed by aspiration with the DCs. Cells were treated in the absence of fetal calf serum (FCS) for the first hour of treatment. Then, the medium was completed to 10% FCS to maintain DC survival during the following 15 h of treatment. Indeed, serum provides a protein concentration, probably higher than what is expected in real exposure, but 10% is necessary for DCs culture. We found it necessary to minimize the impact of serum proteins. Previous work showed that the short one-hour incubation without FCS improved the sensitivity of the assay, allowing us to use lower concentrations, without altering the outcome as the same markers were found expressed. We therefore retained these experimental conditions for the following experiments. In some conditions, moDCs were pre-treated for one hour with Syk inhibitor IV (1 µM), methyl-ß-cyclodextrin (5 mM) or Saracatinib (10 µM). Syk inhibitor IV (BAY 61–3606) and methyl-β-cyclodextrin were purchased from Millipore (Massachussets, USA). Saracatinib was purchased from Selleck Chemicals (Texas, USA).

### Phenotypical analysis

After pre-treatment or not with Syk inhibitor, moDCs were stimulated for 16 h with SAS-NPs and moDC viability and phenotype were evaluated by flow cytometry. Cell viability was assessed in preliminary experiments by propidium iodide (PI) (Invitrogen, California, USA) staining, used at a final concentration of 625 ng.mL^−1^ on a small fraction of moDC cultures. Under these experimental conditions, SAS-NPs did not interfere with the result of the cytotoxicity test. Cell viability after treatment was never less than 70%. For the phenotypic analysis, the surface labeling procedure was as follows: 2 × 10^5^ cells.mL^−1^ were washed in cold phosphate-buffered saline (PBS) supplemented with 0.5% bovine serum albumin (BSA) and stained with monoclonal antibodies (mAbs) in the dark, at 4 °C for 20 min. The following mAbs with appropriate isotype controls were used: CD80^FITC^ (557,226, BD Biosciences, Le Pont de Claix, France), CD83^PE^ (556,855, BD Biosciences), CD86^APC^ (555,660, BD Biosciences). Cells were analysed on an Attune Nxt (Invitrogen) using FlowJo software (version 10; FlowJo LLC, Oregon, USA). We used a gating strategy to exclude dead cells, based on the FSC/SSC criteria. The data acquisition was performed on a minimum of 10,000 living cells. Results were expressed as the relative fluorescence intensity (RFI), using the corrected mean fluorescence intensity (cMFI) as follows: cMFI = MFI-MFI of isotype control; RFI = cMFI of treated cells/cMFI of untreated cells.

### Allogeneic co-cultures of moDC and T cells

CD4^+^ T-cells were isolated from PBMCs by positive selection with MidiMACS separation columns and anti-CD4 antibodies coated on magnetic beads (Miltenyi Biotec). These T-cells were confirmed to have a purity > 95%, based on CD4 expression evaluated by flow cytometry (561,841, RPA-T4, BD Biosciences). CD4^+^ T-lymphocytes were labeled with 0.5 mM carboxyfluorescein succinimidyl ester (CFSE) (Invitrogen), following the manufacturer’s instructions. MoDCs were pre-treated or not with inhibitors and stimulated as previously described, then washed and co-cultured with allogeneic CD4^+^ T-cells at a 1:20 DC/T-cell ratio for 5 days, in RPMI 1640 Glutamax supplemented with 10% human serum (Sigma Aldrich, Missouri, USA) in round-bottom 96-well plates. CFSE fluorescence was analysed using flow cytometry, on an Attune Nxt (Invitrogen) using FlowJo software (version 10; FlowJo LLC). Proliferating T-cells corresponded to low CFSE fluorescence.

### Cytokine and chemokine quantification in the cell culture supernatants

MoDC/CD4^+^ T-cell coculture supernatants were measured in duplicate for IFN-γ, IL-5, IL-9, IL-10, IL-13, IL-17A, IL-17F, and IL-22 using Meso Scale Discovery multiplex assay (MSD, Rockville, MA, USA) following manufacturer’s instructions. The quantification ranges were as follows: IFN-γ, 21.26–87,100 pg.mL^−1^; IL-5, 5.07–20,800 pg.mL^−1^; IL-9, 0.3–1,230 pg.mL^−1^; IL-10, 4.76–19,500 pg.mL^−1^; IL-13, 2.40–9,832 pg.mL^−1^; IL-17A, 29.29–120,000 pg.mL^−1^, IL-17F 155–112,000 pg.mL^−1^and IL-22, 0.83–3,420 pg.mL^−1^.

### Immunoblot analysis

After pre-treatment or not with inhibitors, moDCs were incubated for 30 to 120 min with 12.5 and 25 µg.mL^−1^ SAS-NPs and washed in cold PBS before lysis. Cells were lysed in lysis buffer (40 mM Tris HCl, pH 7,4; 274 mM NaCl; 4 mM EDTA; 2% Triton X-100; 4 mM Sodium pyrophosphate; 20% Glycerol and H_2_O) to which phosphatase and protease inhibitors (Sigma-Aldrich, Saint-Louis, USA) were added (aprotinin 1 mg.mL^−1^; leupeptin 1 mg.mL^−1^; phenylmethylsulfonylfluoride 0,1 M; sodium orthovanadate 100 mM, pepstatin 10 mg.mL^−1^; 2.5% ß-glycerophosphate). 30 µg of denatured proteins were loaded into 12% SDS-PAGE gels (TGX stain-free Fastcast acrylamide kit, Bio-Rad, Marnes-La-Coquette, France) and transferred to nitrocellulose membranes (Bio-Rad), which were successively incubated with antibodies directed against the phosphorylated (phospho-Syk Tyr525/526 antibody, Cell Signalling) and total forms of Syk (Cell Signalling). Membranes were then incubated with phosphoP38, phosphoJNK and ß-actin (Cell signaling). Immunoreactive bands were detected by their chemiluminescence using the ChemiDoc XRS + System (Bio-Rad Laboratories, Marnes-La-Coquette, France). Bands were quantified with ImageLab software.

### Fluorescent staining of lipid rafts and confocal laser scanning microscopy (CLSM)

Lipid rafts were stained using the Cholera Toxin Subunit B, Alexa Fluor 488 Conjugate (Molecular Probes, Invitrogen) after 15 and 30 min of stimulation with SAS-NPs.

The samples were imaged with an inverted confocal laser scanning microscope TCS SP8 –gSTED Leica (Leica, Germany) using a HC PL APO CS2 63 × /1.40 oil immersion objective lens. The instrument was equipped with a WLL Laser (488 nm excitation wavelength). Green fluorescence was collected with a 490–600 nm wide emission slit on a PMT detector. Transmission images were acquired with a PMT-trans detector. The pinhole was set at 1.0 Airy unit giving an optical slice thickness of 0.89 µm. 12-bit numerical images were done with the Leica Application Suite X software (Version 3.5.5; Leica).

### Statistical analysis

Data were expressed as means ± SEM. Differences between groups were analysed using a non-parametric unpaired Kruskal–Wallis test (Prism software; GraphPad, La Jolla, CA). When the data complied with the normality test (Shapiro Wilk test), one-way ANOVA with Bonferroni’s post-test was conducted. The *p* values < 0.05 were considered statistically significant.

## Supplementary Information

**Additional file 1.** **Figure S1. A.** Size distribution of fumed silica nanoparticles before and after sterilization. Intensity-weighted size distribution profiles of fumed silica nanoparticles in pure RPMI 1640, in RPMI 1640 supplemented with 1% of Fetal Calf Serum (FCS) and in RPMI 1640 supplemented with 10% FCS were obtained using Dynamic Light Scattering. Each measure was repeated ten times. **B.** ζ-potential variations of fumed silica nanoparticles in RPMI 1640 with or without fetal calf serum before and after sterilization (ζ-potential data are represented as mean ± SEM of ten independent measurements).

## Data Availability

Not applicable.

## Abbreviations

SAS-NPs: Amorphous silica nanoparticles
DCs: Dendritic cells
moDCs: Monocyte-derived dendritic cells
MHC-II: Major histocompatibility complex class-II
LPS: Lipopolysaccharide
PHA: Phytohaemagglutinin
DLS: Dynamic light scattering
LAL: Limulus amebocyte lysate
PBMCs: Peripheral blood mononuclear cells
PI: Propidium iodide
CFSE: Carboxyfluorescein succinimidyl ester
