# Supplementary figures and images for: Human dendritic cell maturation induced by amorphous silica nanoparticles is Syk-dependent and triggered by lipid raft aggregation
Source: Part Fibre Toxicol. 2023 Apr 19;20:12. doi: 10.1186/s12989-023-00527-9 (PMC10114393; doi:10.1186/s12989-023-00527-9)

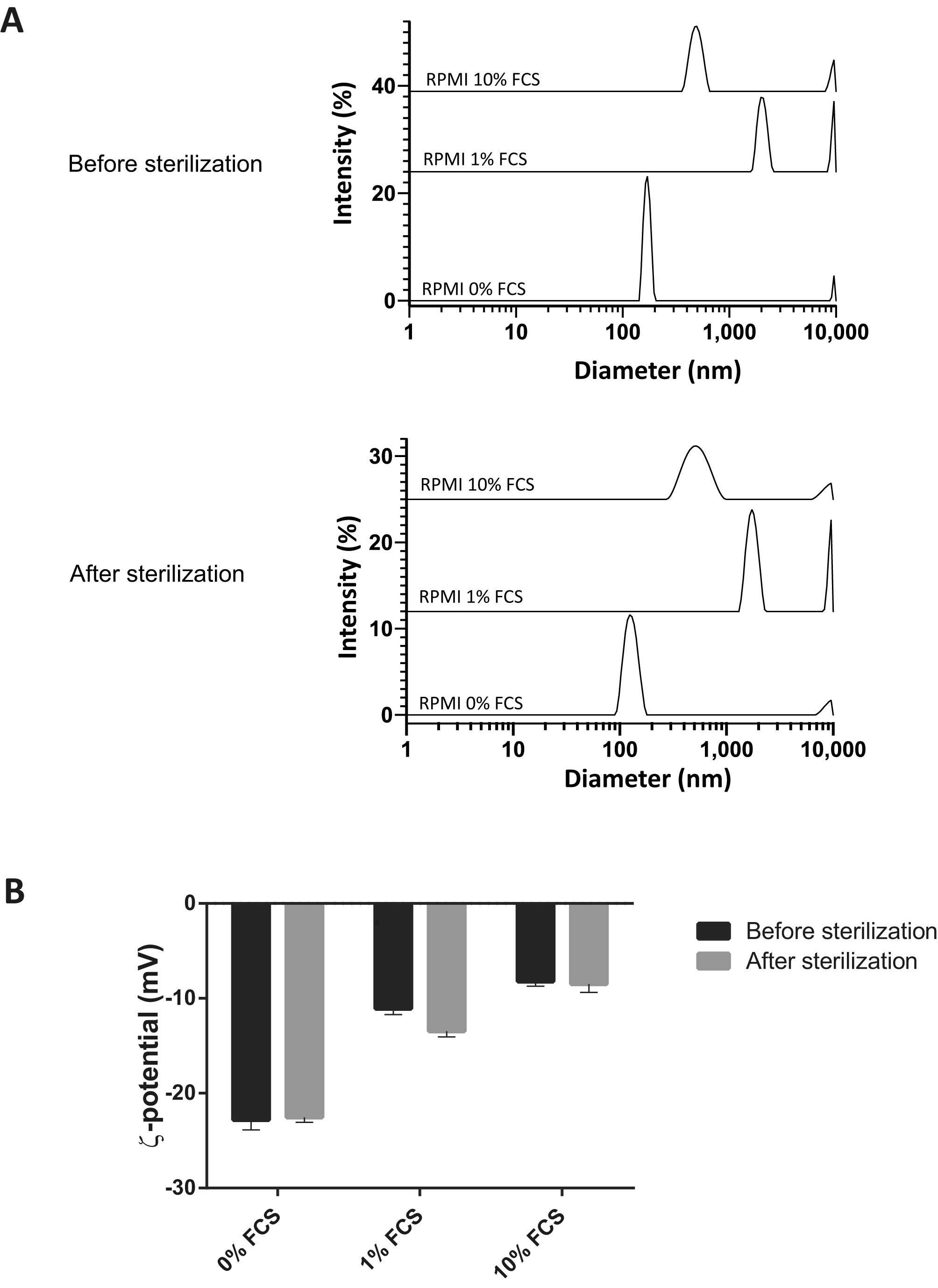

Supplement: Supplementary file 1 — Additional file 1. Figure S1. A. Size distribution of fumed silica nanoparticles before and after sterilization. Intensity-weighted size distribution profiles of fumed silica nanoparticles in pure RPMI 1640, in RPMI 1640 supplemented with 1% of Fetal Calf Serum (FCS) and in RPMI 1640 supplemented with 10% FCS were obtained using Dynamic Light Scattering. Each measure was repeated ten times. B. ζ-potential variations of fumed silica nanoparticles in RPMI 1640 with or without fetal calf serum before and after sterilization (ζ-potential data are represented as mean ± SEM of ten independent measurements). [file 12989_2023_527_MOESM1_ESM.tif]
